# SARS-CoV-2 neutralising antibody testing in Europe: towards harmonisation of neutralising antibody titres for better use of convalescent plasma and comparability of trial data

**DOI:** 10.2807/1560-7917.ES.2021.26.27.2100568

**Published:** 2021-07-08

**Authors:** Dung Nguyen, Peter Simmonds, Maurice Steenhuis, Elise Wouters, Daniel Desmecht, Mutien Garigliany, Marta Romano, Cyril Barbezange, Piet Maes, Bram Van Holm, Joaquín Mendoza, Salvador Oyonarte, Anders Fomsgaard, Ria Lassaunière, Eva Zusinaite, Katarina Resman Rus, Tatjana Avšič-Županc, Johan HJ Reimerink, Fiona Brouwer, Marieke Hoogerwerf, Chantal BEM Reusken, Gunnveig Grodeland, Sophie Le Cam, Pierre Gallian, Abdennour Amroun, Nadège Brisbarre, Christophe Martinaud, Isabelle Leparc Goffart, Hubert Schrezenmeier, Hendrik B Feys, C Ellen van der Schoot, Heli Harvala

**Affiliations:** 1 University of Oxford, Oxford, United Kingdom; 2 Department of Immunopathology, Sanquin Research and Landsteiner Laboratory Academic Medical Centre, Amsterdam, Netherlands; 3 Transfusion Research Centre, Belgian Red Cross-Flanders, Ghent, Belgium; 4 Department of Pathology, Faculty of Veterinary Medicine, Liège University, Liège, Belgium; 5 Immune Response service, Sciensano, Brussels, Belgium; 6 National Influenza Centre, Sciensano, Brussels, Belgium; 7 KU Leuven, Rega Institute, Clinical and Epidemiological Virology, Leuven, Belgium; 8 Vircell SL, Granada, Spain; 9 Andalusian Network of Transfusion Medicine, Tissues and Cells, Sevilla, Spain; 10 Virus and Microbiological Special Diagnostics, Statens Serum Institute, Copenhagen, Denmark; 11 Tartu University Institute of Technology, Tartu, Estonia; 12 Institute of Microbiology and Immunology, Ljubljana, Slovenia; 13 Centre for Infectious Disease Control, WHO COVID-19 Reference Laboratory, RIVML, Bilthoven, the Netherlands; 14 Dep. of Immunology, University of Oslo and Oslo University Hospital, Oslo, Norway; 15 Etablissement Français du Sang, La Plaine Saint Denis, France; 16 Unité des Virus Émergents (Aix-Marseille University – IRD 190 – Inserm 1207 – IHU Méditerranée Infection), Marseille, France; 17 Etablissement français du Sang Provence Alpes Côte d’Azur et Corse, Marseille, France; 18 Centre de Transfusion Sanguine des Armées, Clamart, France; 19 Unité de Virologie, Institut de Recherche Biomédicale des Armées, Marseille, France; 20 Department of Transfusion Medicine, Ulm University, Ulm, Germany; 21 Institute for Clinical Transfusion Medicine and Immunogenetics, German Red Cross Blood Transfusion Service Baden-Wurttemberg – Hessen and University Hospital Ulm, Ulm, Germany; 22 Microbiology Services, NHS Blood and Transplant, Colindale, United Kingdom; 23 University College of London, London, United Kingdom

**Keywords:** SARS-CoV-2, neutralising antibodies, convalescent plasma, Europe, standardisation, COVID-19

## Abstract

We compared the performance of SARS-CoV-2 neutralising antibody testing between 12 European laboratories involved in convalescent plasma trials. Raw titres differed almost 100-fold differences between laboratories when blind-testing 15 plasma samples. Calibration of titres in relation to the reference reagent and standard curve obtained by testing a dilution series reduced the inter-laboratory variability ca 10-fold. The harmonisation of neutralising antibody quantification is a vital step towards determining the protective and therapeutic levels of neutralising antibodies.

Individuals infected with severe acute respiratory syndrome coronavirus 2 (SARS-CoV-2) develop a neutralising antibody response, which is a key component of adaptive immunity and considered a primary mechanism of protection against many vaccine-preventable infections [1-5]. Neutralising antibodies can also be used as therapeutics, via passive transfer of monoclonal or polyclonal antibodies to prevent or cure infections [6-9]. Convalescent plasma containing a sufficient level of neutralising antibodies has been successfully used as a prophylactic or early treatment for SARS-CoV-2 infection [10-12]. However, neutralising antibody testing has not been standardised and hence titres obtained in one study cannot be compared with those obtained in others. Here we describe SARS-CoV-2 live virus neutralisation testing and its standardisation across 12 laboratories in nine European countries in order to harmonise titres applied in convalescent plasma trials and for potential future use.

## Panel of convalescent plasma samples

We constructed and provided for blinded testing a panel of 15 SARS-CoV-2 convalescent plasma samples obtained from six individuals in England; the samples had varying neutralising titres in the in-house live virus neutralising assay and reactivity in the EuroImmune anti-spike IgG ELISA (PerkinElmer, London, United Kingdom) (Table 1). The panel included a 1:10 dilution of research reagent 20/130 obtained from the National Institute for Biological Standards and Control (NIBSC, United Kingdom) which had been assigned a unitage of 1,300 international units (IU)/mL of SARS-CoV-2-neutralising antibodies [12]. A dilution series of a high-titre convalescent plasma sample (Sample 1; dilutions labelled as 1A–1D) calibrated in IU/mL against this research reagent and a negative plasma control in duplicate were also included in this panel (Sample 13).

**Table 1:** Details of samples referred for external evaluation of anti-SARS-CoV-2 neutralising antibody testing (n = 10)

Convalescent plasma sample	Included in the panel	Neutralising antibody titre	EUROImmun S/Co ratio	Date of sampling
12	Twice	80	4.32	15 Aug 2020
7	Twice	160	5.27	31 Jul 2020
2	Twice	320	6.72	15 May 2020
19	Twice	640	5.90	26 Nov 2020
13	Twice	< 20	0.12	2 Sep 2020
1A (neat)	Once	5,120	7.06	3 Aug 2020
1B (1:10)	Once	320	3.86	3 Aug 2020
1C (1:50)	Once	80	1.12	3 Aug 2020
1D (1:100)	Once	40	0.11	3 Aug 2020
NIBSC 1:10 (130 IU/mL)^a^	Once	160	1.94	NA

IU: international units; NIBSC: National Institute for Biological Standards and Control; SARS-CoV-2: severe acute respiratory syndrome coronavirus 2; S/Co: signal to cut-off ratio.

^a ^NIBSC research reagent code 20/130.

## Participant blood establishments and laboratories

On 25 February 2021, we sent an email invitation to join this study to 19 blood establishments and associated laboratories involved in SUPPORT-E project (https://www.support-e.eu). The reference panel constructed at our laboratory L*ref *in England (Table 1) was sent to 11 laboratories in eight countries who responded positively to our invitation (Table 2). These included one laboratory each in Denmark, Estonia, the Netherlands, Norway, Slovenia and Spain, three laboratories in Belgium and two in France. Samples were tested by their respective in-house live virus microneutralisation assay against the non-variant SARS-CoV-2 strain (n = 12 laboratories), Alpha isolates (Phylogenetic Assignment of Named Global Outbreak (Pango) lineage designation B.1.1.7; n = 3) or Beta isolates (lineage B.1.351; n = 1) following their normal laboratory practises. Results were expressed as end-point titres (n = 3) or as extrapolated median 50% tissue culture infectious dose (TCID_50_) values (n = 9). 

**Table 2:** Details for live SARS-CoV-2 microneutralisation assays performed in 96-well plates, included in this study, Europe, April–May 2021 (n = 12 laboratories)

Laboratory	Cell line^a^	Cells per well	When were cells added to the 96-well plate?	Virus per well	MOI^b^	Length of incubation	Assay read-out	Assay cut-off titre	Virus lineage
**L*ref***	VERO E6	10,000	After incubation of plasma with virus	50 TCID50	0.005	4 days at 37 °C	CPE	< 1:40	B (D614)
**L2**	VERO E6	20,000	After incubation of plasma with virus	100 TCID50	0.005	5 days at 37 °C	CPE	< 1:20	B.1 (D614)
**L3**	VERO E6	15,000^c^	Preseeded 72 h before experiment	100 TCID50	0.00667	5 days at 37 °C	CPE	< 1:20	B.1 (G614)
**L4**	VERO E6	16,000	Preseeded 24 h before experiment	300 TCID50	0.01875	24 h at 37 °C	N-protein ELISA^d^	< 1:10	B.1.1 (G614)
**L6**	VERO E6	20,000^c^	Preseeded 24 h before experiment	100 TCID50	0.005	48 h at 37 °C	N-protein ELISA^d^	< 1:40	B.1.1.5 (G614)
**L8**	VERO E6	40,000	After incubation of plasma with virus	100 PFU	0.0025	4 days at 37 °C	CPE	< 1:4	B.1.1 (G614)
**L9**	VERO E6	10,000	Preseeded 24 h before experiment	100 TCID50	0.01	5 days at 37 °C	CPE	< 1:20	B.1.1 (G614)
**L10**	VERO E6	20,000	After incubation of plasma with virus	100 TCID50	0.005	3 days at 35 °C	CPE	< 1:10	B.1.1 (G614)
**L11**	VERO E6	10,000	Preseeded 24 h before experiment	100 TCID50	0.01	3 days at 37 °C	N-protein ELISA^d^	< 1.0	B (D614)
**L12**	VERO	16,000	After incubation of plasma with virus	100 TCID50	0.00625	4 days at 37 °C	CPE	< 1:20	B.1.153 (G614)
**L13**	VERO E6	20,000	After incubation of plasma with virus	400 PFU	0.02	5 days at 37 °C	CPE	< 1:50	A (D614)
**L14**	VERO E6	50,000	Preseeded 24 h before experiment	100 TCID50	0.002	5 days at 37 °C	CPE	< 1:20	B.1.153 (G614)

CPE: cytopathic effect; MOI: multiplicity of infection; TCID50: median tissue culture infectious dose; PFU: plaque-forming units; TICD50: median tissue culture infectious dose.

^a^ All cell lines obtained from the American Type Culture Collection (Manassas, United States) and free from mycoplasma contamination: VERO E6 #CRL-1586 and VERO #CCL81.

^b^ MOI was calculated based on the amount of virus (TCID50 or PFU) used for infection.

^c^ Cell count at the time of seeding.

^d^ In-house ELISA where commercially obtained antibody against the nucleocapsid is used to quantify the virus replication.

## Detection of neutralising antibodies

To assess assay specificity, all 12 laboratories measured virus-neutralising antibody titres for two replicates of the anti-SARS-CoV-2-negative sample against the non-variant SARS-CoV-2 strain (Sample 13). Nine laboratories reported neutralisation titres below the negative cut-off range; however, L12 reported low titres in both replicates and two laboratories (L8 and L13) reported low/medium titres in both replicates, indicating possible specificity issues in ca a quarter of participating laboratories.

## Linearity of neutralising antibody testing

**Figure 1:**
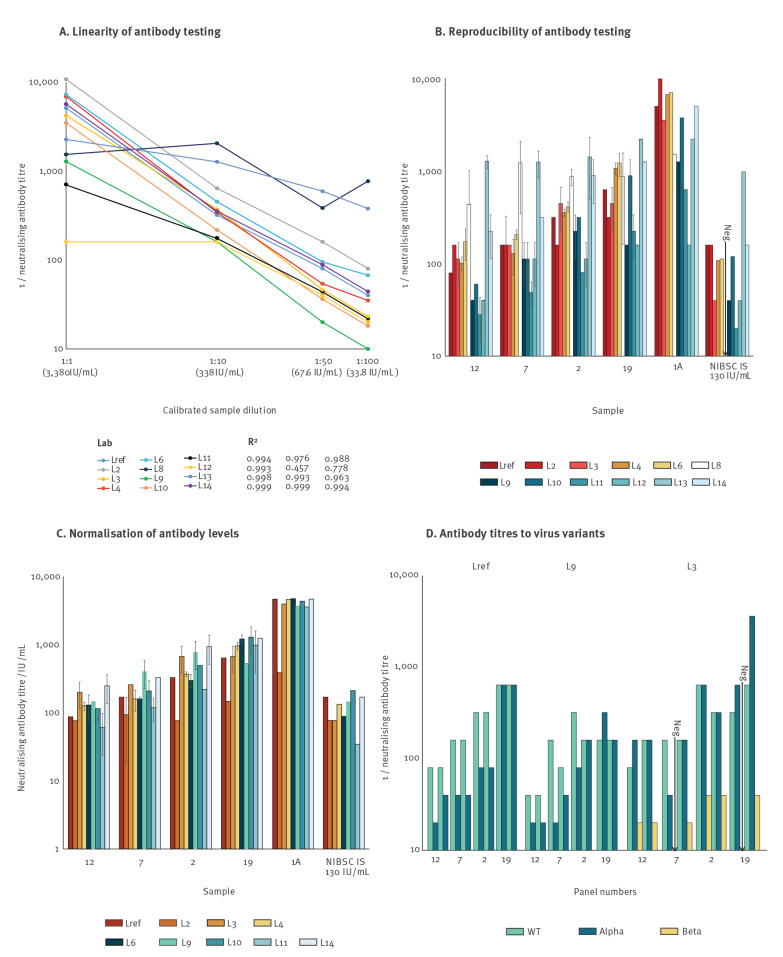
Neutralising antibody titres for SARS-CoV-2 in the plasma sample panel, Europe, April–May 2021 (n = 12 laboratories)

IS: internal standard; IU: international unit; NIBSC: National Institute for Biological Standards and Control; SARS-CoV-2: severe acute respiratory syndrome coronavirus 2; WT: non-variant SARS-CoV-2 strain.

Linearity of neutralising antibody quantification was assessed by testing a serial dilution of a high-titre anti-SARS-CoV-2 sample, calibrated in IU/mL (Samples 1A-1D). Linear regression was used to assess correlation coefficients of log-transformed plasma dilutions and antibody titres. Neutralisation titres showed a linear relationship with dilution in the results from nine of the 12 laboratories and very high correlation coefficients (R^2^ > 0.975) between log transformed values (Figure 1A), even though the absolute titres reported by the laboratories varied over a 10-fold range. Results from one laboratory (L12) showed non-linearity for the undiluted, highest titre sample, L13 showed a non-linear relationship between titre and dilution even though the correlation co-efficient was high (R^2^ = 0.96), while L8 showed little relationship between titre and dilution (R^2^ = 0.46) (Supplementary Table S1). Two of the laboratories used a smaller multiplicity of infection (describing the amount of virus used to infect a single cell: 0.0025 for L8 and 0.002 for L14) than most other laboratories.

A. Linearity of neutralising antibody testing assessed by testing a dilution series (Samples 1A–1D). For results expressed as end-point dilutions, any overlapping graph lines were minimally offset to separate the depicted titres.

B. Neutralising antibody titres for convalescent samples included in the panel; bar heights show geometric means for Samples 12, 7, 2 and 19 tested in two replicates per sample and error bars show standard deviations.

C. Normalised neutralising antibody levels after conversion to IU/mL. Neutralisation titres from each assay were converted to IU/mL using linear regression formulae derived from assay calibration with the pre-quantified control shown in panel A. The conversion used derived assay-specific multipliers and constants listed in Supplementary Table S1. Error bars show standard deviations. The poor correlation of reported antibody titres with dilution of the pre-quantified control reported by L8, L12 and L13 precluded their inclusion in the depiction of normalised antibody levels.

D. Neutralising antibody titres assessed with SARS-CoV-2 Alpha (B.1.1.7) and Beta (B.1.351) variants.

Neutralising antibody titres on positive plasma samples provided in duplicate (Samples 12, 7, 2 and 19) showed moderate reproducibility between replicates (Figure 1B). The remaining results showed a wide range of titres that may have arisen, at least in part, from different testing methods as presented in Table 2; for example, assay titres reported by L13 were consistently higher than those reported by other laboratories, while those from L11 were consistently lower. Variability between results reported by different laboratories were often large; L11 reported a neutralising antibody titre of 1:20/1:40 to sample 12 whereas L13 reported a titre 1:1,455/1:1,165 to this same sample.

## Normalisation of neutralising antibody level measurements

**Figure 2:**
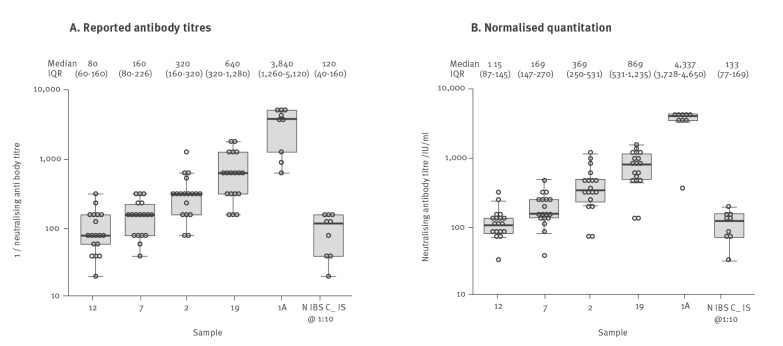
Range of reported raw neutralising antibody titres and normalised titres, Europe, April–May 2021 (n = 12 laboratories)

IQR: interquartile range; NIBSC_IS: National Institute for Biological Standards and Control internal standard; SARS-CoV-2: severe acute respiratory syndrome coronavirus 2.

The neutralising antibody results were calibrated in relation to a standard curve obtained by testing of a dilution series calibrated in IU/mL. Neutralisation titres from each assay were converted to IU/mL using linear regression formulae derived from assay calibration with the pre-quantified control as shown in Figure 1A. The conversion used derived assay-specific multipliers and constants listed in Supplementary Table S1. Results from L8 and L12 were excluded from normalisation because they lacked linearity, and L13 was excluded for non-proportionality on testing the dilution series. Normalised quantitation of antibody levels showed moderate reproducibility between replicates (Figure 1C) and more than 10-fold reduced inter-laboratory variability compared with raw titres (Figure 2). Furthermore, testing of the 1:10 diluted NIBSC reagent yielded a median neutralising antibody concentration of 133 IU/mL after normalisation, which was close to the expected 130 IU/mL value and also demonstrated low variability between assays (interquartile range: 87–169, < 2-fold). 

The laboratories tested a panel of four convalescent plasma samples in duplicate, one in singular and an NIBSC research reagent at a concentration of 130 IU/mL. Results from L8, L12 and L13 have been excluded from normalisation the results lacked linearity and these data are therefore not included in panel A.

## Neutralising antibody titres against SARS-CoV-2 variants

Three participating laboratories (L*ref*, L3 and L9) reported neutralisation titres with the SARS-CoV-2 variant Alpha (lineage B.1.1.7) and one laboratory (L3) with variant Beta (lineage B.1.351). For Samples 12, 7, 2 and 19, antibody titres were typically lower against Alpha strains than against non-variant virus (Figure 1D); these differences were less marked in L*ref* and L9 than in L3. Reported neutralising antibody levels against the antigenically more distinct Beta strain were substantially lower than against non-variant SARS-CoV-2. 

## Ethical statement

All samples were obtained via the non-clinical services from NHS Blood and Transplant, England. Signed donor consent was obtained for purposes of clinical audit and research, as well as to assess and improve the services including diagnostic testing.

## Discussion

Our analysis demonstrates that normalisation and standardisation using internal controls is achievable for virus neutralisation assays employing a wide range of formats. It should be used to support not only the harmonisation of antibody concentrations present in convalescent plasma supplied for clinical use and trials but also for the assessment of its efficacy. Our data also demonstrated clearly that neutralising antibody levels in our panel samples were generally lower against Beta strains than against non-variant SARS-CoV-2 isolates. These data are consistent with previously published observations that the spike mutations in Beta, and to a lesser extent also in Alpha, variants are associated with reduced susceptibility to neutralising antibodies [13-15]. Therefore, these must be considered in data analysis, especially when SARS-CoV-2 variants have emerged during the convalescent plasma trial periods [11,16,17]. Further international standards and harmonisations are required for each SARS-CoV-2 variant of concern.

It is a limitation of our study that we used plasma samples only and that we did not compare the microneutralisation assay with a plaque reduction neutralisation test (PRNT) which has been shown to be more sensitive in detecting neutralising antibodies [18]. As all participating laboratories had introduced a microneutralisation assay for convalescent plasma testing, we felt that evaluation of PRNT assay or serum samples was less relevant in this study. However, it is important to note the potential differences in neutralising antibody titres obtained by PRNT and microneutralisation assay can also be overcome by harmonisation of those readings into IU/mL.

## Conclusions

Our data demonstrate substantial heterogeneity in neutralising antibody testing used to determine antibody content in convalescent plasma donations across Europe. This is not surprising as this gold standard method was mostly used as a research tool before the COVID-19 pandemic and has only recently been subjected to standardisation [12]. Although we observed almost 100-fold differences in raw neutralising antibody titres between the participating laboratories, these readings could largely be harmonised by adopting the NIBSC international standard. As many convalescent plasma trials have either been previously reported or are approaching their endpoints, a large meta-analysis is urgently required that focuses on identifying the efficacy of convalescent plasma based on neutralising antibody dose. The harmonisation and quantification of neutralising antibody testing is a first step towards determining a cut-off for protective and therapeutic levels of neutralising antibodies present in convalescent plasma. It is also an essential tool when using neutralising antibody data to assess and compare vaccine effectiveness from different SARS-CoV-2 vaccine candidates and clinical trials. 

## Supplementary Data

171000 Supplement
