# Supplementary material for: SARS-CoV-2 neutralising antibody testing in Europe: towards harmonisation of neutralising antibody titres for better use of convalescent plasma and comparability of trial data
Source: Euro Surveill. 2021 Jul 8;26(27):2100568. doi: 10.2807/1560-7917.ES.2021.26.27.2100568 (PMC8268650; doi:10.2807/1560-7917.ES.2021.26.27.2100568)
Supplement: Supplement [file 21-00568_HARVALA_Supplement.pdf]

SUPPLEMENTARY DATA

Table S1

Correlation coefficients and normalisation formulae derived from neutralising antibody testing of a serial dilution of high-titre anti-SARS-CoV-2 sample by 12 laboratories.

**Lab code**	**R2**[1]	***p***	**m**[2]	**c**[2]
Lref	0.994	0.003	-1.05	12.15
L2	0.993	0.066	-1.07	13.49
L3	0.998	0.001	-1.14	11.91
L4	0.999	<0.001	-1.22	12.32
L6	0.976	0.098	-0.96	12.27
L8	0.457[4]	0.324	-0.25	10.91
L9	0.993	0.003	-1.07	10.49
L10	0.999	<0.001	-1.15	11.84
L11	0.988	0.006	-0.75	9.49
L12	0.778	0.118	-0.45	7.82
L13	0.963	0.019	-0.38	11.29
L14	0.994	0.003	-1.05	12.15

[1]Pearson correlation coefficients for log transformed values from the calibrated sample dilution series.

[2,3]Muliplier and constant in equations for each regression: y = mx + c

[4]Problematic results for correlation coefficients or linearity are shown in red and led to their exclusion from normalisation analysis.

This supplementary material is hosted by Eurosurveillance as supporting information alongside the article [SARS-CoV-2 neutralising antibody testing in Europe: towards harmonisation of neutralising antibody titres for better use of convalescent plasma and comparability of trial data], on behalf of the authors, who remain responsible for the accuracy and appropriateness of the content. The same standards for ethics, copyright, attributions and permissions as for the article apply. Supplements are not edited by Eurosurveillance and the journal is not responsible for the maintenance of any links or email addresses provided therein.
